# The Involvement of Proteoglycans in the Human Plasma Prekallikrein Interaction with the Cell Surface

**DOI:** 10.1371/journal.pone.0091280

**Published:** 2014-03-12

**Authors:** Camila Lopes Veronez, Fabio D. Nascimento, Katia R. B. Melo, Helena B. Nader, Ivarne L. S. Tersariol, Guacyara Motta

**Affiliations:** 1 Departamento de Bioquímica, Escola Paulista de Medicina/UNIFESP, São Paulo, Brazil; 2 Biomaterials and Biotechnology Research Group - UNIBAN, São Paulo, Brazil; National Cancer Institute, NIH, United States of America

## Abstract

**Introduction:**

The aim of this work was to evaluate the role of human plasma prekallikrein assembly and processing in cells and to determine whether proteoglycans, along with high molecular weight kininogen (H-kininogen), influence this interaction.

**Methods:**

We used the endothelial cell line ECV304 and the epithelial cell lines CHO-K1 (wild type) and CHO-745 (deficient in proteoglycans). Prekallikrein endocytosis was studied using confocal microscopy, and prekallikrein cleavage/activation was determined by immunoblotting using an antibody directed to the prekallikrein sequence C_364_TTKTSTR_371_ and an antibody directed to the entire H-kininogen molecule.

**Results:**

At 37°C, prekallikrein endocytosis was assessed in the absence and presence of exogenously applied H-kininogen and found to be 1,418.4±0.010 and 1,070.3±0.001 pixels/cell, respectively, for ECV304 and 1,319.1±0.003 and 631.3±0.001 pixels/cell, respectively, for CHO-K1. No prekallikrein internalization was observed in CHO-745 in either condition. Prekallikrein colocalized with LysoTracker in the absence and presence of exogenous H-kininogen at levels of 76.0% and 88.5%, respectively, for ECV304 and at levels of 40.7% and 57.0%, respectively, for CHO-K1. After assembly on the cell surface, a plasma kallikrein fragment of 53 kDa was predominant in the incubation buffer of all the cell lines studied, indicating specific proteolysis; plasma kallikrein fragments of 48–44 kDa and 34–32 kDa were also detected in the incubation buffer, indicating non-specific cleavage. Bradykinin free H-kininogen internalization was not detected in CHO-K1 or CHO-745 cells at 37°C.

**Conclusion:**

The prekallikrein interaction with the cell surface is temperature-dependent and independent of exogenously applied H-kininogen, which results in prekallikrein endocytosis promoted by proteoglycans. Prekallikrein proteolysis/activation is influenced by H-kininogen/glycosaminoglycans assembly and controls plasma kallikrein activity.

## Introduction

The plasma kallikrein/kinin system, which comprises the contact system proteins plasma prekallikrein, high molecular weight kininogen (H-kininogen) and Factor (F)XII is a physiologic mediator of vascular biology and inflammatory reactions. Human plasma kallikrein is a protease that was first found to affect hemostasis by amplifying FXII activation and inflammation by H-kininogen hydrolysis and bradykinin release. Plasma kallikrein also affects fibrinolysis and plasmin formation by single-chain urokinase plasminogen activation or plasminogen cleavage [1]. Other functions have also been attributed to plasma kallikrein, such as the activation of the plasminogen cascade in adipogenesis and mammary gland development [2], [3], tissue repair and angiogenesis through the hepatocyte growth factor/c-Met activation pathway [4], [5], and hepatic regeneration by latent TGF-β activation [6].

The plasma kallikrein-kinin system proteins have been implicated in the pathogenesis of inflammation, hypertension, endotoxemia, coagulopathy, angiogenesis, epithelial cell apoptosis, adipocyte differentiation, and stromal remodeling and the interaction with cell surface could be a mechanism for controlling their activities [7].

The H-kininogen interaction with the surfaces of endothelial cells is mediated by a protein complex involving the globular head domains of the complement component C1q, the urokinase plasminogen activator receptor, and cytokeratin 1 [8]. Proteoglycans may function as binding sites for H-kininogen and promote its internalization [9], [10], [11] [12]. Heparin released from activated mast cells triggers edema during allergic reactions and inflammatory diseases by activating the coagulation intrinsic pathway [13]. It is well known that H-kininogen is a potent proangiogenic molecule through bradykinin release. On the other hand, plasma kallikrein cleaved H-kininogen (bradykinin free H-kininogen) is a potent antiangiogenic agent [14]. Taken together, besides regulating VEGF-VEGFR signaling system [15], [16], [17] cell surface proteoglycans can also regulate angiogenesis by modulating plasma kallikrein-kinin system activity.

In our previous work, we showed that human plasma prekallikrein, the zymogen form of plasma kallikrein, specifically and reversibly binds to human umbilical vein endothelial cells (HUVECs) in the presence or absence of exogenously applied H-kininogen. The cell-associated plasma prekallikrein is then rapidly activated to plasma kallikrein independently of exogenous FXII [18]. Because cell-bound H-kininogen is cleaved by mature plasma kallikrein on HUVECs, bradykinin can be released near the endothelium where it exerts its functions and bradykinin free H-kininogen can be generated [19].

Cerf *et al*. [20] observed intense labeling for plasma kallikrein in endothelial cells, foamy macrophages, inflammatory cells and fibroblasts within the thickened intima of the plaque as well as in smooth muscle cells of the underlying tunica media. The authors suggested the possibility of plasma kallikrein induction by atheromatous disease. Fink *et al*. [21] demonstrated prekallikrein or plasma kallikrein localization in various human tissues including single cells of the distal tubules in nephrons, hepatic epithelial cells, cells of the pancreatic islet of Langerhans, interstitial Leydig cells of the testes, follicular and thecal granulosa cells of the ovary, the parotid gland, the esophagus, skin, the respiratory tract, the prostate and the breast. In addition, Chee *et al*. [22] found plasma kallikrein in the cytoplasm and demonstrated nuclear labeling of malignant mesothelioma cells.

Plasma kallikrein-kinin proteins have also been found on the surface and in the extracellular matrix of endothelial cells from line ECV304 [23], vascular smooth muscle cells [24], immortalized human EA.hy926 endothelial cells [25], lung epithelial cells [26] and endothelial cells from a line derived from rabbit aorta RAECs [27]. Little is known regarding the mechanisms of plasma prekallikrein interactions with cells. The direct prekallikrein interaction with vascular smooth muscle cells has shown possible prekallikrein activation in the absence of an exogenous source of H-kininogen [28].

In the present work, we examined prekallikrein assembly and processing on cells and analyzed whether glycosaminoglycans (GAGs), along with H-kininogen, influence this interaction. We used the vascular cell line, ECV304, as our endothelial cell model, and exogenous prekallikrein was internalized either in the absence or presence of exogenously applied H-kininogen. We used wild-type Chinese hamster ovarian cells (CHO-K1) and mutant CHO cells deficient in PG biosynthesis (CHO-745). We found that prekallikrein is taken up and directed to endosomes/lysosomes and that this endocytosis process is mediated by GAGs. In addition, prekallikrein may be cleaved in the presence or absence of GAGs. Therefore, prekallikrein activation while in contact with H-kininogen on the cell surface is influenced by GAGs.

## Materials and Methods

### Material

All chemicals obtained from commercial sources were of the best grade available. The biotinylation kit (NHS-LC-Biotin), Immunopure streptavidin horseradish peroxidase conjugated (SA-HRP), SuperSignal West Pico Chemiluminescent Substrate and the Dylight 659 Protein Labeling kit were purchased from Pierce Biotechnology Inc. (Rockford, IL, USA). Prekallikrein and H-kininogen were purchased from EMD Biosciences, Inc. (La Jolla, CA,USA); bradykinin free H-kininogen was purchased from Enzyme Research Laboratories (South Bend, IN, USA). Lyso Tracker (LT) Red DND-99 and Green and 4′-6-diamidino-2-phenylindole dihydrochloride (DAPI) were obtained from Molecular Probes/Invitrogen Detection Technologies (Eugene, OR, USA). Prestained Broad Range or Low Range molecular weight standards were purchased from Bio-Rad Laboratories Ltd. (Hercules, CA, USA). Membrane Immobilon-P transfer membrane (PVDF) was obtained from Millipore Corporation (Billerica, MA, USA). Fluoromont-G was purchased from Electron Microscopy Sciences (Hatfield, PA, USA). FITC-conjugated streptavidin was obtained from Jackson ImmunoResearch Laboratories Inc. (West Grove, PA, USA). Rabbit anti-human plasma kallikrein IgG (U-691.10) was produced by the commercial service Eurogentec (Liège, Belgium) and goat anti-rabbit IgG peroxidase conjugated was obtained from Sigma-Aldrich Co. (St. Louis, MO, USA). Rabbit anti-human H-kininogen IgG was produced following the protocol established by our group [29] and the serum was purified on Immobilized Protein A obtained from Thermo Scientific, Rockford, IL, USA). Plasma kallikrein was purified in our laboratory following a previously described protocol [30].

### Cell lines and culture conditions

All cell lines used in this study were approved by the Ethics Committee on Research of EPM/UNIFESP (CEP 1207/10). ECV304, a cell line established from primary culture of HUVECs [31], [32], was purchased from American Type Culture Collection (ATCC). The epithelial cell lines CHO-K1 and CHO-745 (the mutant deficient in xylosyltransferase) [33] were gifts from Dr. Jeffrey D. Esko (Department of Cellular and Molecular Medicine, Glycobiology Research & Training Center, University of California). All cell lines were cultured in a humidified incubator containing 2.5% CO_2_ at 37°C and subcultured in Ham F-12 nutrient mixture medium supplemented with 10% (v/v) heat-inactivated fetal calf serum containing 10 U penicillin and 10 μg/ml streptomycin and grown to confluence on 60 mm dishes.

### Biotinylation of prekallikrein and labeling of bradykinin free H-kininogen with a fluorescent dye

Prekallikrein was biotinylated (biotin-prekallikrein) as previously reported [18], and Dylight 659-bradykinin free H-kininogen was prepared following the manufacturer's instructions. After labeling, the remaining free fluorescent dye was eliminated by gel filtration in a PD-10 column obtained from GE Healthcare UK Ltd. The degree of labeling was estimated by absorbance measurements as indicated by the dye manufacturer.

### Immunocytochemistry

Cells were cultured on 13.0 mm diameter glass coverslips (5×10^3^ cells/coverslip) in 24 well plates for 2 days. In the analysis of prekallikrein internalization, the medium was removed, the cells were washed five times with incubation buffer (Ham F-12 medium without serum containing 50 μM Zn^2+^), and LT Red DND-99 (0.5 μM diluted in incubation buffer) was added for 20 min at 37°C for labeling the endocytic compartments. In the experiments performed at 4°C, after the incubation with LT Red, the cells were maintained at 4°C for an additional 30 min. After this step, the cells were washed three times with incubation buffer and incubated with H-kininogen (100 nM) or biotin-prekallikrein (100 nM) at 4°C or 37°C. The unbound H-kininogen was removed by washing the cells three times with incubation buffer, and then the cells were incubated with biotin-prekallikrein (100 nM). Biotin-prekallikrein was removed by aspiration, and the cells were washed with 10 mM sodium phosphate and 150 mM NaCl at pH 7.4 (PBS) and fixed with 2% (v/v) paraformaldehyde in PBS (pH 7.4) for 20 min at room temperature. The cells were washed four times with PBS and once with PBS containing 0.1 M glycine. They were then permeabilized with PBS containing 1% (w/v) BSA and 0.01% (w/v) saponin for 15 min at room temperature. After this step, the cells were washed with PBS and incubated with FITC-conjugated streptavidin [5 μg/ml PBS containing 0.01% (w/v) saponin] for 1 h at room temperature. After washing, the cells were incubated with DAPI (2 μg/ml) in PBS containing 0.01% (w/v) saponin for 20 min at room temperature. The cells were washed and mounted in Fluoromount-G diluted in PBS (2∶1, v/v) and examined using a scanning confocal microscope. Each figure shown in the results section corresponds to the best of two experiments.

Time-lapse confocal fluorescence images taken under a confocal laser scanning microscope were used to examine the cellular trafficking of bradykinin free H-kininogen (5–30 min). For imaging, living CHO cells grown on coverslips were washed with incubation buffer and pre-incubated with 0.5 μM LT Green DND-99 diluted in incubation buffer at 37°C for 20 min for labeling the endocytic compartments. The cells were then washed with incubation buffer and incubated with Dylight 659-bradykinin free H-kininogen (200 nM) diluted in incubation buffer. The fluorescent signals of LT Green DND-99 and Dylight 659-bradykinin free H-kininogen (red) as well as the phase contrast micrographs were monitored in real time at 37°C with a confocal laser scanning microscope every 5 min. The labeled molecules in acidic compartments were analyzed using an inverted confocal laser-scanning microscope (Zeiss LSM-510, Carl Zeiss, Jena, Germany). FITC-streptavidin and LT Green were visualized under a 488-nm excitation from a CW Argon ion laser and its emission was detected at 490-550 nm (green channel). LT Red and Dylight 659 were observed with HeNe2 laser with excitation at 633 nm and fluorescence emission was detected at 640–670 nm (red channel). Nuclear compartments were stained with DAPI and visualized under 359-nm excitation from multiphoton and its emission was detected at 450–470 nm. Cells were also imaged by differential interference contrast microscopy (DIC), using a HeNe2 laser at 633 nm. False color fluorescent images and transmitted light images (512 × 512 pixels) were stored, quantified and managed using the resident LSM Image Browser software (Carl Zeiss, Jena, Germany). This allowed fluorescence intensity profiles and colocalization rates to be measured, thereby providing mean (±SD) intensity values for green and red emission channels of each image.

### Prekallikrein processing after assembly in different cell lines

Experiments were performed to analyze whether the prekallikrein that assembled on the cell surface, with or without exogenous H-kininogen, was processed. Cells grown in 96 microtiter plate wells were incubated with H-kininogen (100 nM) or prekallikrein (100 nM) in incubation buffer at 37°C for 1 h (ECV304) or 30 min (CHO cells), and unbound H-kininogen was removed by aspiration. The cells were then incubated at 37°C with prekallikrein (100 nM), and the incubation buffer from four replicates of each time point (total volume of 400 μl) containing the unbound prekallikrein was combined and lyophilized; the cells were washed with PBS and solubilized by adding electrophoresis sample buffer (10 μl) containing 3% (v/v) β-mercaptoethanol. The replicate samples were combined (total volume of 40 μl), boiled, separated by SDS-PAGE [13% (v/v)], electroblotted onto PVDF membrane and blocked with PBS containing 0.01% (v/v) Tween 20 and 5% (w/v) non-fat milk. Anti-H-kininogen or anti-plasma kallikrein (U-691.10) directed to the prekallikrein sequence C_364_TTKTSTR_371_, which is exposed after prekallikrein cleavage and activation [21], was added, and the complexes were detected by goat anti-rabbit IgG peroxidase (HRP) conjugated. The antigen/primary antibody/secondary antibody-HRP complexes were detected using SuperSignal substrate. Each figure shown corresponds to the best experiment among at least two replicates.

## Results

### Biotin-prekallikrein endocytosis is mediated by proteoglycans

**Figure 1:**
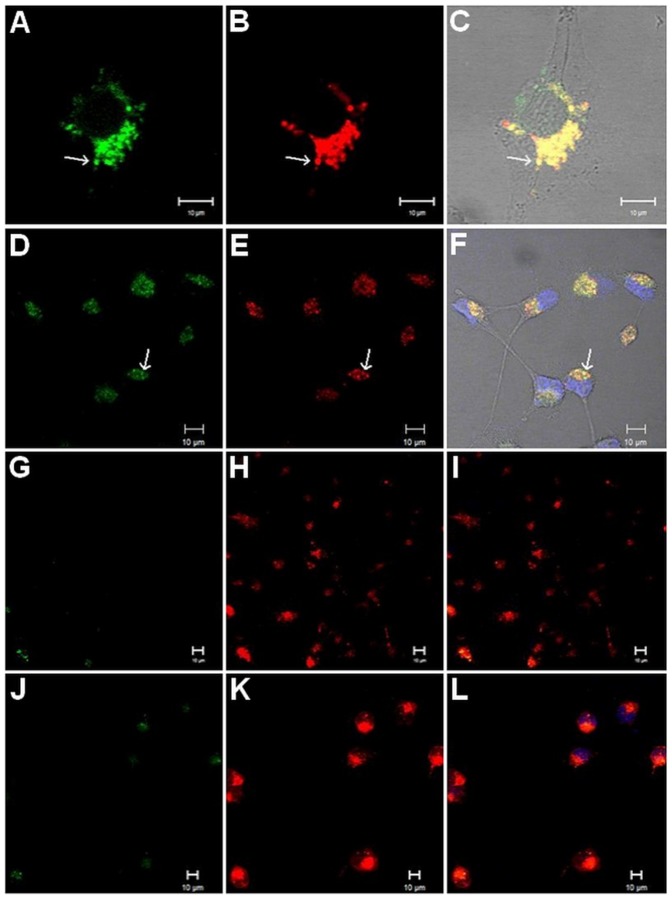
Endocytosis of biotin-prekallikrein by ECV304 cells. ECV304 cells were grown on cover slips, and the lysosomes/endosomes were labeled with 0.5 μM LT Red DND-99 in incubation buffer for 20 min at 37°C. They were treated with or without H-kininogen (100 nM, unlabelled) for 1 h and then treated with biotin-prekallikrein (100 nM) for 1 h at 37°C. The cells were incubated with FITC-conjugated streptavidin (green). Alternatively, after labeling with 0.5 μM LT Red DND-99 at 37°C, the cells were maintained at 4°C and then treated with or without H-kininogen (100 nM, unlabelled) for 1 h and with biotin-prekallikrein (100 nM) for 1 h at 4°C. Biotin-prekallikrein (green) endocytosis and intracellular localization are indicated by their colocalization with acidic vesicles previously labeled with LT Red DND-99 (red) and were analyzed by confocal fluorescence microscopy. Normal endocytosis by ECV304 cells at 37°C without H-kininogen (A–C): biotin-prekallikrein (A), lysosomes/endosomes labeled with LT Red DND-99 (B), merged images and diphasic contrast (C); with H-kininogen (D–F): biotin-prekallikrein (D), lysosomes/endosomes labeled with LT Red DND-99 (E), merged images and diphasic contrast (F). Normal endocytosis by ECV304 at 4°C without H-kininogen (G–I): biotin-prekallikrein (G), lysosomes/endosomes labeled with LT Red DND-99 (H), merged images and diphasic contrast (I); with H-kininogen (J–L): biotin-prekallikrein (J), lysosomes/endosomes labeled with LT Red DND-99 (K), merged images and diphasic contrast (L).

The interaction between biotin-prekallikrein and ECV304 cells was investigated with and without exogenous H-kininogen. For ECV304 at 37°C, Figure 1 (A–C) shows internalization without H-kininogen (1,418.4±0.010 pixels/cell; 76.0% prekallikrein colocalized with LT), and Figure 1 (D–F) shows prekallikrein internalization with H-kininogen (1,070.3±0.001 pixels/cell; 88.5% prekallikrein colocalized with LT). The experiments were also performed at 4°C to prevent endocytosis, and Figure 1 (G–I) shows almost no prekallikrein internalization without H-kininogen (124.0±0.0 pixels/cell; 90.3% prekallikrein colocalized with LT). Similarly, Figure 1 (J–L) shows no prekallikrein internalization with H-kininogen (69.0±0.001 pixels/cell; 93.7% colocalized with LT). Interestingly, prekallikrein was internalized into ECV304 cells and colocalized with LT in acidic endosomal vesicles regardless of the presence of H-kininogen.

Because heparan sulfate and chondroitin sulfate proteoglycans accumulate with H-kininogen on the cell surface [9], [10] and because H-kininogen internalization is mediated by heparan sulfate [12], we examined the possibility that prekallikrein endocytosis is mediated by proteoglycans using the cell lines CHO-K1 and CHO-745.

**Figure 2:**
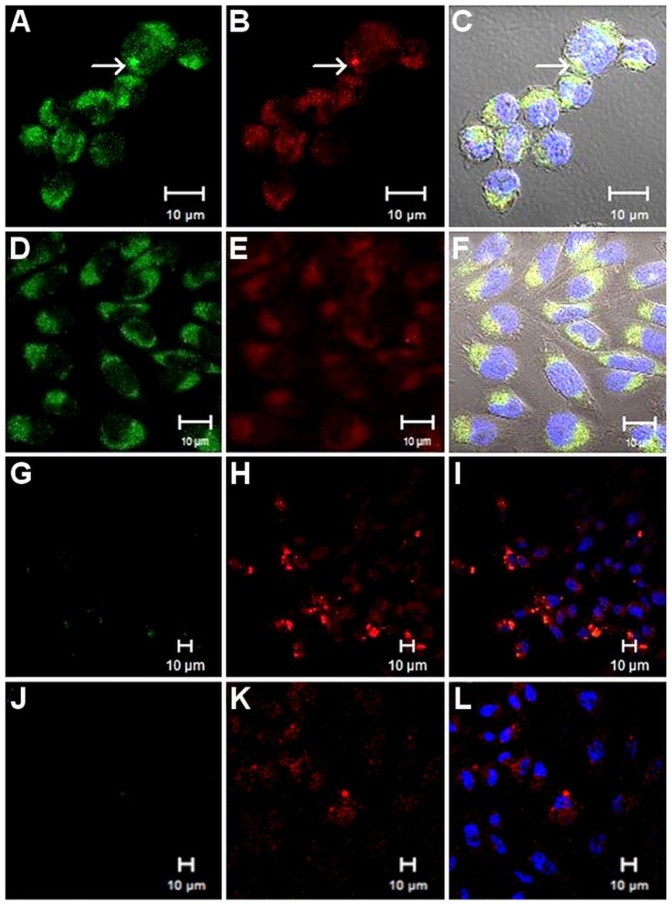
Endocytosis of biotin-prekallikrein by CHO-K1 cells. CHO-K1 cells were grown on cover slips, and the lysosomes/endosomes were labeled with 0.5 μM LT Red DND-99 in incubation buffer for 20 min at 37°C. They were then treated with or without H-kininogen (100 nM, unlabelled) for 30 min and then with biotin-prekallikrein (100 nM) for 1 h at 37°C. The cells were incubated with FITC-conjugated streptavidin (green). Alternatively, after labeling with 0.5 μM LT Red DND-99 at 37°C, the cells were maintained at 4°C and then treated with or without H-kininogen (100 nM, unlabelled) for 30 min and with biotin-prekallikrein (100 nM) for 1 h at 4°C. Biotin-prekallikrein (green) endocytosis and intracellular localization are indicated by their colocalization with acidic vesicles previously labeled with LT Red DND-99 (red) and were analyzed by confocal fluorescence microscopy. Normal endocytosis by CHO-K1 cells at 37°C without H-kininogen (A–C): biotin-prekalikrein (A), lysosomes/endosomes labeled with LT Red DND-99 (B), merged images and diphasic contrast (C); with H-kininogen (D–F): biotin-prekalikrein (D), lysosomes/endosomes labeled with LT Red DND-99 (E), merged images and diphasic contrast (F). Normal endocytosis by CHO-K1 cells at 4°C without H-kininogen (G–I): biotin-prekallikrein (G), lysosomes/endosomes labeled with LT Red DND-99 (H), merged images and diphasic contrast (I); with H-kininogen (J–L): biotin-prekallikrein (J), lysosomes/endosomes labeled with LT Red DND-99 (K), merged images and diphasic contrast (L).

At 37°C, prekallikrein was internalized without H-kininogen in the CHO-K1 line (Figure 2 A–C) (1,319.1±0.003 pixels/cell; 40.7% of prekallikrein colocalized with LT); nevertheless, prekallikrein internalization was lower with H-kininogen (631.3±0.001 pixels/cell; 57.0% prekallikrein colocalized with LT) (Figure 2 D–F). The experiments performed at 4°C with CHO-K1 confirmed that prekallikrein endocytosis depends on temperature because no prekallikrein was detected inside the cells (0.0±0.001 pixels/cell) with and without exogenous H-kininogen Figures 2 (G–L).

**Figure 3:**
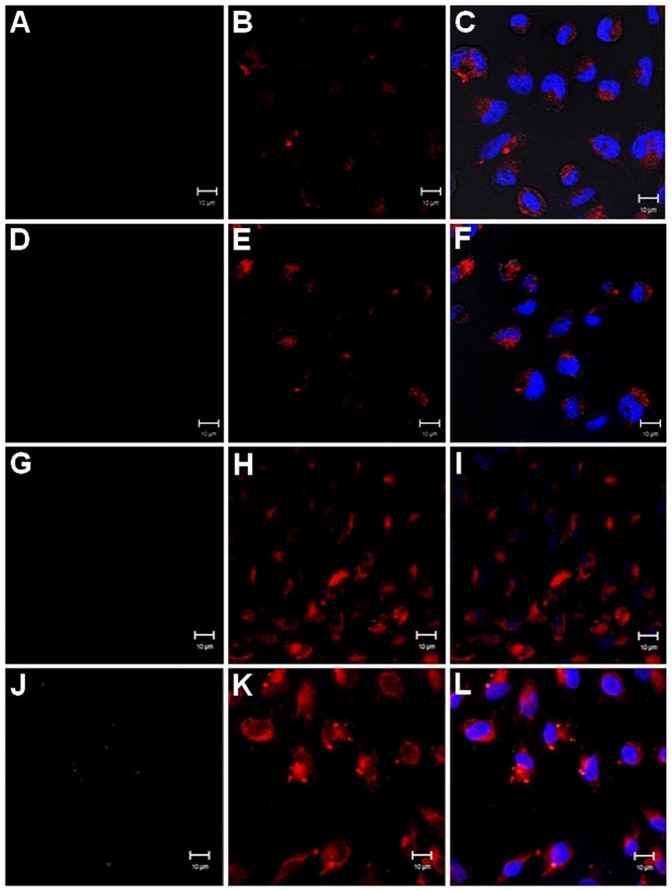
Endocytosis of biotin-prekallikrein by CHO-745 cells. CHO-745 cells were grown on cover slips, and the lysosomes/endosomes were labeled with 0.5 μM LT Red DND-99 in incubation buffer for 20 min at 37°C. Then the cells were treated with or without H-kininogen (100 nM, unlabelled) for 30 min and with biotin-prekallikrein (100 nM) for 1 h at 37°C. The cells were incubated with FITC-conjugated streptavidin (green). Alternatively, after labeling with 0.5 μM LT Red DND-99 at 37°C, the cells were maintained at 4°C and treated with or without H-kininogen (100 nM, unlabelled) for 30 min and with biotin-prekallikrein (100 nM) for 1 h at 4°C. Biotin-prekallikrein (green) endocytosis and intracellular localization are indicated by colocalization with acidic vesicles previously labeled with LT Red DND-99 (red) and were analyzed by confocal fluorescence microscopy. Normal endocytosis by CHO-745 cells at 37°C without H-kininogen (A–C): biotin-prekallikrein (A), lysosomes/endosomes labeled with LT Red DND-99 (B), merged images and diphasic contrast (C); with H-kininogen (D–F): biotin-prekallikrein (D), lysosomes/endosomes labeled with LT Red DND-99 (E), merged images and diphasic contrast (F). Normal endocytosis by CHO-745 cells at 4°C without H-kininogen (G–I): biotin-prekallikrein (G), lysosomes/endosomes labeled with LT Red DND-99 (H), merged images and diphasic contrast (I); with H-kininogen (J–L): biotin-prekallikrein (J), lysosomes/endosomes labeled with LT Red DND-99 (K), merged images and diphasic contrast (L).

The experiments performed using CHO-745 cells at 37°C show that both without and with exogenous H-kininogen, no prekallikrein internalization occurs (0.7±0.002 and 1.6±0.003 pixels/cell, respectively) (Figure 3 (A–F)); CHO-745 cells at 4°C also show no prekallikrein uptake with or without H-kininogen (0.8±0.001 and 4.4±0.001 pixels/cell, respectively) (Figure 3 (G–L)).

### Prekallikrein activation and H-kininogen processing after assembly in various cell lines

GAGs participate in a variety of biological processes including cell-matrix interactions and the activation of chemokines, enzymes and growth factors [34]. We analyzed prekallikrein activation during its uptake and endocytosis at 37°C in the absence and presence of exogenous H-kininogen using the antibody U691.10 to detect fragments that contain the sequence C_364_TTKTSTR_371_, which is present in zymogen prekallikrein. This sequence represents the C-terminal end of the heavy chain of plasma kallikrein, which is newly formed upon activation of prekallikrein by cleavage of the R_371_-I_372_ peptide bond [1], [21].

**Figure 4:**
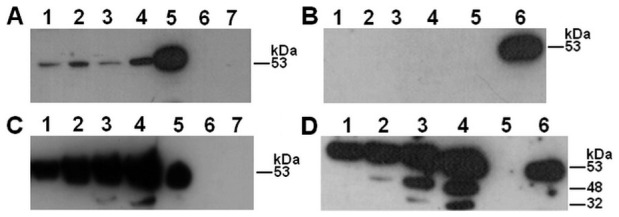
Prekallikrein structure over time after interaction with ECV304 cells. H-kininogen (100 nM) was incubated for 1 h with ECV304 cells, and after removing unbound H-kininogen by aspiration, prekallikrein (100 nM) was added for 15 min, 30 min, 1 h or 2 h at 37°C. Alternatively, prekallikrein was added to the cells at different time points in the absence of assembled H-kininogen. At the end of each time point, the incubation buffer was collected, and both the incubation buffer and the cell samples were treated with reducing sample buffer. Immunoblotting was performed with anti-plasma kallikrein U-691.10 for detecting plasma kallikrein fragments containing the sequence C_364_TTKTSTR_371_. (A) Cells with H-kininogen: (1) 15 min; (2) 30 min; (3) 1 h; (4) 2 h; (5) plasma kallikrein (0.9 μg); (6) prekallikrein (1.6 μg); (7) H-kininogen (0.7 μg). (B) Cells without H-kininogen: (1) 15 min; (2) 30 min; (3) 1 h; (4) 2 h; (5) prekallikrein (1.6 μg); (6) plasma kallikrein (0.9 μg). (C) Incubation buffer with H-kininogen: (1) 15 min; (2) 30 min; (3) 1 h; (4) 2 h; (5) plasma kallikrein (0.9 μg); (6) prekallikrein (1.6 μg); (7) H-kininogen (0.7 μg). (D) Incubation buffer without H-kininogen: (1) 15 min; (2) 30 min; (3) 1 h; (4) 2 h; (5) prekallikrein (1.6 μg); (6) plasma kallikrein (0.9 μg)

In ECV304 cells (Figure 4), prekallikrein cleavage was detected from the cell surface samples with H-kininogen (Figure 4A), as a 53 kDa fragment appeared after a 15 min incubation. This band remained over time and may correspond to the plasma kallikrein heavy chain, indicating prekallikrein proteolysis. Without H-kininogen (Figure 4B), no plasma kallikrein fragments were detected, and the results were very similar to those of experiments performed with HUVECs [18]. The supernatants of the samples with (Figure 4C) and without (Figure 4D) H-kininogen at the cell surface resulted in fragments of 53 kDa and smaller, indicating prekallikrein proteolysis.

**Figure 5:**
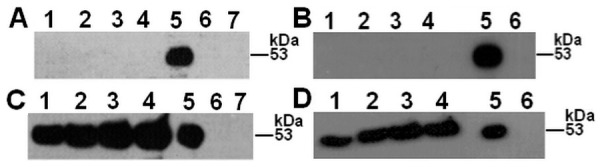
Prekallikrein structure over time after interaction with CHO-K1 cells. H-kininogen (100 nM) was incubated for 30 min with CHO-K1, and after removing H-kininogen by aspiration, prekallikrein (100 nM) was added for 15 min, 30 min, 1 h or 2 h at 37°C. Alternatively, prekallikrein was added to cells at different time points in the absence of assembled H-kininogen. At the end of each time point, the incubation buffer was collected, and both the incubation buffer and the cell samples were treated with reducing sample buffer. Immunoblotting was performed with anti-plasma kallikrein U691.10 for detecting plasma kallikrein fragments containing the sequence C_364_TTKTSTR_371_. (A) Cells with H-kininogen: (1) 15 min; (2) 30 min; (3) 1 h; (4) 2 h; (5) plasma kallikrein (0.9 μg); (6) prekallikrein (1.6 μg); (7) H-kininogen (0.7 μg). (B) Cells without H-kininogen: (1) 15 min; (2) 30 min; (3) 1 h; (4) 2 h; (5) plasma kallikrein (0.9 μg); (6) prekallikrein (1.6 μg). (C) Incubation buffer with H-kininogen: (1) 15 min; (2) 30 min; (3) 1 h; (4) 2 h; (5) plasma kallikrein (0.9 μg); (6) prekallikrein (1.6 μg); (7) H-kininogen (0.7 μg). (D) Incubation buffer without H-kininogen: (1) 15 min; (2) 30 min; (3) 1 h; (4) 2 h; (5) plasma kallikrein (0.9 μg); (6) prekallikrein (1.6 μg)

**Figure 6:**
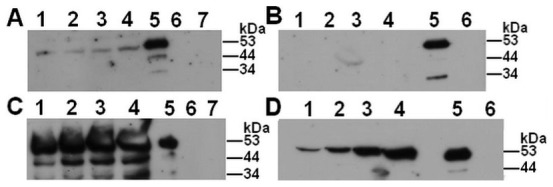
Prekallikrein structure over time after interaction with CHO-745 cells. H-kininogen (100 nM) was incubated for 30 min with CHO-745 cells, and after removing H-kininogen by aspiration, prekallikrein (100 nM) was added for 15 min, 30 min, 1 h or 2 h at 37°C. Alternatively, prekallikrein was added to the cells at different time points in the absence of assembled H-kininogen. At the end of each time point, the incubation buffer was collected, and both the incubation buffer and the cell samples were treated with reducing sample buffer. Immunoblotting was performed with anti-plasma kallikrein U691.10 for detecting plasma kallikrein fragments containing the sequence C_364_TTKTSTR_371_. (A) Cells with H-kininogen: (1) 15 min; (2) 30 min; (3) 1 h; (4) 2 h; (5) plasma kallikrein (0.9 μg); (6) prekallikrein (1.6 μg); (7) H-kininogen (0.7 μg). (B) Cells without H-kininogen: (1) 15 min; (2) 30 min; (3) 1 h; (4) 2 h; (5) plasma kallikrein (0.9 μg); (6) prekallikrein (1.6 μg). (C) Incubation buffer with H-kininogen: (1) 15 min; (2) 30 min; (3) 1 h; (4) 2 h; (5) plasma kallikrein (0.9 μg); (6) prekallikrein (1.6 μg); (7) H-kininogen (0.7 μg). (D) Incubation buffer without H-kininogen: (1) 15 min; (2) 30 min; (3) 1 h; (4) 2 h; (5) plasma kallikrein (0.9 μg); (6) prekallikrein (1.6 μg)

In CHO-K1 cells, either with or without exogenous H-kininogen (Figure 5), no bands were detected over time from the cell surface samples (Figures 5A and 5B). However, in the supernatants of the samples, a 53 kDa fragment can be seen from 15 min to 2 h of incubation with (Figure 5C) and without (Figure 5D) H-kininogen. Although the CHO-745 cells did not internalize prekallikrein, its structure was analyzed after H-kininogen assembly in these cells (Figure 6). Regardless of H-kininogen treatment (Figures 6A and 6B), almost no prekallikrein fragments were detected on the cell surfaces. Surprisingly, in the supernatants of the samples with added H-kininogen, prekallikrein fragments of 53 kDa and smaller were detected by U691.10 starting at a15 min incubation and increasing in intensity over time (Figure 6C). Without H-kininogen, only a prekallikrein fragment of 53 kDa was detected, which also increased in intensity over time (Figure 6D).

Our results clearly showed prekallikrein cleavage/activation independently of GAG; nevertheless, in the presence of H-kininogen assembled on the cell surface through GAG, prekallikrein hydrolysis to plasma kallikrein is more specific.

**Figure 7:**
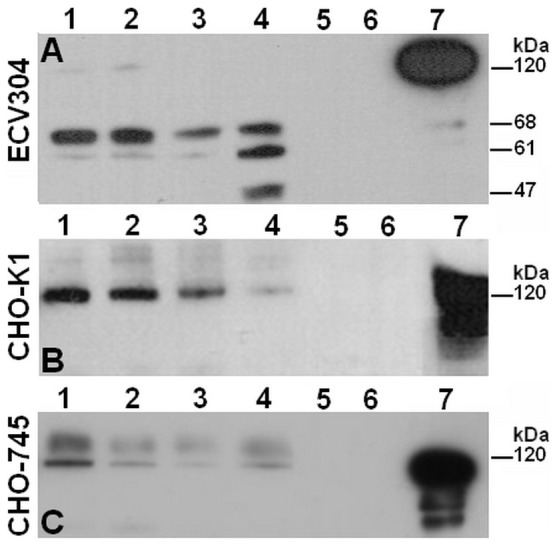
Hydrolysis of cell-associated H-kininogen by mature plasma kallikrein. H-kininogen (100 nM) in the presence of incubation buffer was incubated for 1 h or 30 min, respectively, with ECV304, CHO-K1 and CHO-745 cells at 37°C. After removing H-kininogen by aspiration, prekallikrein (100 nM) was added for 15 min, 30 min, 1 h or 2 h at 37°C. At the end of each time point, the incubation buffer was collected, and both the incubation buffer and the cell samples were treated with reducing sample buffer. Immunoblotting of the cells was performed with the anti-H-kininogen antibody. (A) ECV304: (1) 15 min; (2) 30 min; (3) 1 h; (4) 2 h; (5) plasma kallikrein (0.9 μg); (6) prekallikrein (1.6 μg); (7) H-kininogen (0.7 μg). (B) CHO-K1: (1) 15 min; (2) 30 min; (3) 1 h; (4) 2 h; (5) plasma kallikrein (0.9 μg); (6) prekallikrein (1.6 μg); (7) H-kininogen (0.7 μg). (C) CHO-745: (1) 15 min; (2) 30 min; (3) 1 h; (4) 2 h; (5) plasma kallikrein (0.9 μg); (6) prekallikrein (1.6 μg); (7) H-kininogen (0.7 μg)

We also analyzed the H-kininogen structure assembled on the cell surface after the interaction with prekallikrein (Figure 7). In ECV304 cells (Figure 7A), H-kininogen (120 kDa) cleavage during prekallikrein activation resulted in bands of 68 kDa, 61 kDa and 47 kDa; the same pattern was previously obtained in HUVECs [18]. Therefore, in CHO-K1 cells (Figure 7B) and CHO-745 cells (Figure 7C) H-kininogen remained intact at 120 kDa and based on these results and previous work H-kininogen internalization by CHO cells is only a possibility [9], [10], [11], [12].

**Figure 8:**
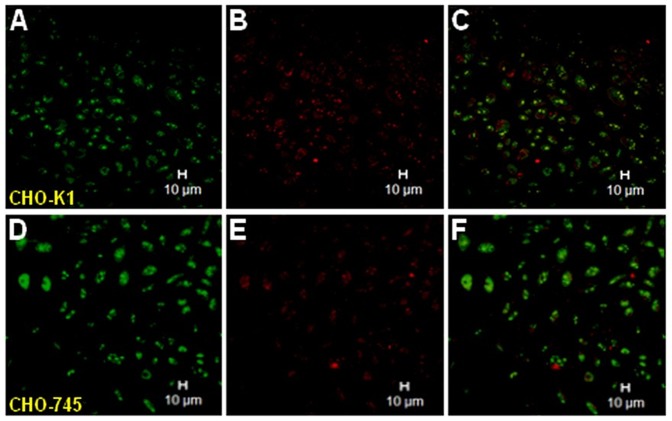
Endocytosis of fluorescent Dylight 659-bradykinin free H-kininogen by CHO cells. CHO-K1 and CHO-745 cells were grown on cover slips, and the lysosomes/endosomes were labeled with 0.5 μM LT Green in Ham F-12 serum-free medium containing 50.0 μM Zn^2+^ for 20 min at 37°C and analyzed by confocal fluorescence microscopy. Dylight 659-bradykinin free H-kininogen (200 nM, red) endocytosis and intracellular localization are indicated by the colocalization with acidic vesicles labeled with LT Green. Normal endocytosis by CHO-K1: (A) LT Green; (B) Dylight 659- bradykinin free H-kininogen; (C) merged images and diphasic contrast. Normal endocytosis by CHO-745: (D) LT Green; (F) Dylight 659- bradykinin free H-kininogen; (G) merged images and diphasic contrast.

We next examined the possibility that bradykinin free H-kininogen is also internalized by GAGs. The endocytic compartments of living CHO cells were labeled with LT Green DND-99 at 37°C for 30 min, after which Dylight 659-bradykinin free H-kininogen (200 nM) was added to the cells. We used confocal fluorescence images to examine the localization of bradykinin free H-kininogen at shorter endocytosis times (5–30 min). As seen in Figure 8, Dylight 659-bradykinin free H-kininogen (red) and LT Green DND-99 fluorescence did not colocalize within endosomal acidic vesicles either in CHO-K1 (49.9±0.002 pixels/cell, Figure 8A–C) or CHO-745 cells (34.7±0.001 pixels/cell, Figure 8D–F).

## Discussion

This work shows that the endocytosis of prekallikrein/plasma kallikrein depends on proteoglycans or GAGs but not on H-kininogen. These results clearly show that (i) the prekallikrein interaction with cells is temperature-dependent, (ii) prekallikrein endocytosis is promoted by proteoglycans or GAGs and (iii) prekallikrein may interact directly with the cell surface independently of H-kininogen but dependent on the GAG composition of the cell type.

In ECV304 cells (Figure 1), the data support our previous work performed in HUVECs [18] that showed that prekallikrein binding occurs in the absence of exogenously applied H-kininogen. This result suggests a lower affinity specific binding site(s) present on these cells. At 37°C, using CHO-K1 (Figure 2) and CHO-745 (Figure 3) cells, we showed that proteoglycans or GAGs mediate biotin-prekallikrein endocytosis. The amounts of heparan sulfate and chondroitin sulfate produced by CHO-745 cells correspond, respectively, to 1% and 2.16% of those produced by CHO-K1 cells [35]. Our results strongly suggest that proteoglycans or GAGs on the cell surface function as additional putative receptors for prekallikrein and that they direct prekallikrein to acidic endosomal vesicles, as we showed previously for the H-kininogen interaction with cells [12]. Fink *et al.* have shown the prekallikrein/plasma kallikrein cellular localization in the cytoplasm and on the nuclear envelope in multiple different progenitor derived cells indicating specific cellular functions of this enzyme that for instance resides in the endoplasmic reticulum of particular cells, in addition to its known function in the blood [21]. The authors attributed to prekallikrein gene transcription in non-hepatic tissues but our results show at the first time that prekallikrein/plasma kallikrein can be internalized by interaction with proteoglycans on cell surface.

In this work, we analyzed the prekallikrein structure upon its interaction with the cell surface to investigate whether prekallikrein cleavage/activation is influenced by interaction with proteoglycans or GAGs or H-kininogen. Prekallikrein hydrolysis was assessed by detecting the protein bands containing the sequence C_364_TTKTSTR_371_, using the antibody U691.10, which is present in plasma kallikrein after prekallikrein cleavage/activation [21]. In ECV304 and CHO-745 cells, a prekallikrein fragment of 53 kDa was detected that was bound to the cell surface in assembled H-kininogen complexes. However, in CHO-K1 cells this band was not detected and also in all cell lines studied without exogenously applied H-kininogen. In ECV304 cells (Figure 4A) the results are in agreement with our previous work using HUVECs since a 53 kDa band is detected after exogenous H-kininogen and prekallikrein assembly on cell surface indicating plasma kallikrein formation [18]. Considering CHO-745 cells (Figure 6A) a weak 53 kDa band corresponding to prekallikrein/plasma kallikrein fragment is detected on cell surface with H-kininogen, probably due to some exogenous H-kininogen bound to other binding sites different from GAGs on cell surface [8]. Therefore, in CHO-K1 cells (Figure 5A) the 53 kDa band corresponding to prekallikrein fragment was not detected with exogenously applied H-kininogen because the ternary complex GAG/H-kininogen/prekallikrein formed on cell surface protected prekallikrein from hydrolysis and active plasma kallikrein formation was avoided.

The results in the fluid phase (incubation buffer) are interesting because the 53 kDa fragment is present in both the presence and absence of H-kininogen. However, in CHO-745 cells prekallikrein proteolysis occurs at different sites (Figure 6C), likely due to the H-kininogen bound to sites on cell surface different from GAG and smaller fragments are also seen for the incubation buffer from ECV304 treated with and without H-kininogen (Figures 4C and 4D, respectively).

Indeed it is well known that GAGs interact with H-kininogen at the endothelial cell surface [9], [11], [27], as well as GAGs also interact with plasma kallikrein modifying its kinetics behavior [36], [37]. Prekallikrein interaction with cells may generate plasma kallikrein by proteolytic cleavage and its activity can be analyzed by H-kininogen structure. In ECV304 cell surface the H-kininogen fragments of 68 kDa, 61 kDa and 47 kDa (Figure 7A) suggest a plasma kallikrein formed by one peptide bond cleavage on prekallikrein (R_371_-I_372_) [1]. Therefore, in CHO-K1 (wild type) we conclude that on cell surface the ternary complex GAG/H-kininogen/prekallikrein protects prekallikrein from hydrolysis (Figure 5A) and intact H-kininogen internalization is only possibility (Figure 7B) [9], [10], [11], [12]. Interestingly in these cells prekallikrein is internalized more in absence of exogenous H-kininogen (Figure 2). Considering CHO-745 (mutant deficient in GAGs) a weak 53 kDa band indicating a plasma kallikrein fragment can be detected on cell surface (Figure 6A), therefore H-kininogen remains intact showing none H-kininogen fragment lower than 120 kDa on cell surface (Figure 7C). Probably the plasma kallikrein formed on CHO-745 surface is cleaved on heavy chain because plasma kallikrein fragments of 53 kDa, 44 kDa and 34 kDa are detected in incubation buffer (Figure 6C). In our previous report [38], we also detected plasma kallikrein bands of 43 kDa, 30 kDa and 20 kDa in activated plasma, which may correspond to the plasma kallikrein cleaved heavy chain. It has been described that an intact heavy chain on prekallikrein/plasma kallikrein structure is very important for the perfect interaction between prekallikrein and H-kininogen [39] which improves bradykinin release by plasma kallikrein [40], [41].

Our results clearly show that prekallikrein interaction with GAGs does not disturb its cleavage/activation (Figure 5D). In fact, H-kininogen bound to GAG may modulate prekallikrein cleavage/activation as a control mechanism of prekallikrein/plasma kallikrein activity (Figures 5A and 5C). Moreover, Renné *et al.* have shown that bradykinin formation is controlled by H-kininogen binding to and detachment from GAGs at the endothelial cells [25].

Since H-kininogen is cleaved on cell surface releasing bradykinin [18], [19] the bradykinin free H-kininogen interaction with cell surface was also examined and the results (Figure 8) show that neither in presence (CHO-K1) nor absence (CHO-745) of GAGs bradykinin free H-kininogen endocytosis is detected suggesting that mature bradykinin free H-kininogen remains outside the cells where it exerts its regulation of angiogenesis [42].

Our results indicate that plasma kallikrein proteolytic balance of H-kininogen/bradykinin free H-kininogen forms is controlled by GAGs modulating the production of bradykinin at endothelial cell surface [18], [25]. GAGs may promote this control by two different ways controlling either the proteolytic activity of the ternary complex GAG/plasma kallikrein/H-kininogen (Figures 5, 6, 7 and 8) or the cellular compartmentalization of kallikrein-kinin system proteins via endocytosis (Figures 2 and 3). The prekallikrein/plasma kallikrein endocytosis mediated by proteoglycans or GAGs may explain plasma kallikrein localization to the connective tissue-type mast cells in the mammary gland and other tissues [21], [2].

In conclusion, our experiments analyzing prekallikrein after interactions with the cell surface either in the presence or absence of exogenously applied H-kininogen show that prekallikrein endocytosis and proteolysis are mechanisms that contribute independently to the control of plasma kallikrein activity and are mediated by proteoglycans.
